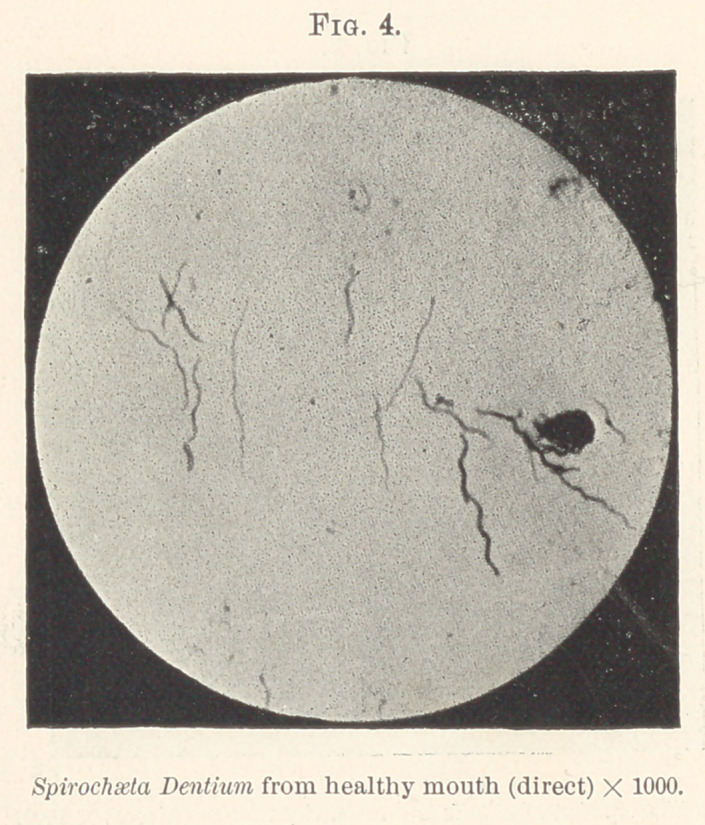# Some Points in Connection with the Bacteria of the Mouth

**Published:** 1896-10

**Authors:** J. W. Washbourn, K. W. Goadby


### Abstracts and Translations.

### SOME POINTS IN CONNECTION WITH THE BACTERIA OF THE MOUTH.[1]

BY J. W. WASHBOURN, M.D., F.R.C.P., AND K. W. GOADBY.

A KNOWLEDGE of the nature and functions of the bacteria found in the mouth is of the greatest importance both to dental surgeons and to medical men. To the dental surgeon it is of importance on account of the *rôle* played by bacteria in the production of caries and of various local affections, while to the medical man it is of importance in the study of the etiology of many infective processes, such, for instance, as diphtheria or scarlet fever.

We have been especially interested in the relation of the streptococci found in the mouth to those occurring in septicæmic conditions in the human subject. We have made a number of observations in this connection, and we venture, with some diffidence, on account of their incomplete nature, to bring our results before you to-night.

In the course of our investigations we have examined the mouths of a large number of healthy and sick individuals, so that we have obtained a fairly extensive practical knowledge of the bacteria of the mouth.

Any one who has studied this question will be struck with certain

[1] Transactions of the Odontological Society of Great Britain.

facts. In the first place, bacteria are found in all mouths, whether the teeth are sound or carious, and whether the individual is well or ill. Secondly, when the teeth are carious, there are generally many more bacteria present in the mouth than when the teeth are sound. Thirdly, in acute diseases more bacteria are present than in health. Lastly, a systematic cleansing of the teeth with the tooth-brush greatly diminishes the number of the bacteria present. An example which came under our notice well illustrates this point. We made a series of examinations of the mouth of a boy with sound teeth, on account of the number of spirilla which were constantly present. One day, to our surprise, the spirilla had completely disappeared, and on inquiry it turned out that the boy had taken to the use of a tooth-brush, as he was getting tired of the repeated examinations.

Cleansing of the mouth diminishes the number of bacteria, partly mechanically, and partly by removing *débris* of food and dead epithelial cells upon which the bacteria flourish. All the conditions which favor the retention of particles of food, such, for instance, as a close packing of the teeth, also favor the growth of bacteria.

An overgrowth of bacteria in the mouth is checked by certain natural processes. First, the saliva acts not only by mechanically removing the bacteria, but also in virtue of its bactericidal properties. Sanarelli has shown that fresh saliva destroys some bacteria and hinders the growth of others. In this respect it is similar to the blood serum and to other fluids of the body. Secondly, the cells which are contained in the lymphoid tissue of the tonsils act as phagocytes, englobing and destroying bacteria.

The importance of diminishing the number of bacteria in the mouth is due to the undoubted fact that caries is caused by their agency. It is well known that caries occurs most frequently in those who neglect their teeth. There are, no doubt, other causes which favor the production of caries, such as an imperfect development of the teeth, a deficient calcification, and so on; but these are only predisposing causes, and without the agency of bacteria caries does not occur.

The exact manner in which caries is produced appears to be the following: Bacteria multiply in various parts of the teeth where cracks or irregularities allow of the collection of particles of food. During their growth acid is formed and a decalcification takes place. The organic matrix of the tooth thus becomes exposed, and then serves as food for the bacteria; and a further formation of acid occurs, which again decalcifies fresh portions of the tooth. The bacteria penetrate into the healthy structure through the dentinal tubes, and produce a lateral destruction of the elastin walls of the tubes and ultimately of the surrounding tissue, so that a large cavity may be formed with only a small external opening. Should the pulp cavity be reached, inflammation occurs and suppuration may ensue.

Caries must not be considered a specific process due to only one kind of bacterium. There are many species of bacteria which will produce caries, just as there are many kinds of bacteria which will produce inflammation and suppuration in various parts of the body.

We should only weary you by attempting to give a list of the species of bacteria that have been found in the mouth at one time or another by different observers. Many of these bacteria are only occasional visitors which have been introduced with the food or air. They remain for a short time in the mouth and then disappear, the conditions not being favorable for their development. When we consider the large number of species of bacteria contained in ordinary drinking water, it is no cause for surprise that systematic cultivations made from the mouth reveal the presence of many kinds of bacteria.

But apart from the bacteria that are, so to speak, accidentally present, the mouth contains certain species which constitute its normal flora. Some of the constant inhabitants of the mouth appear to be incapable of multiplying outside the body under the ordinary conditions of nature, and, indeed, some species have resisted all attempts at cultivation in artificial media. Some, on the other hand, can be cultivated in the various media generally employed.

On examining the different regions of the mouth certain species of bacteria are met with most frequently in certain localities. This localization is most apparent when the teeth are sound and the mouth systematically cleansed.

Scrapings from the mucous membrane of the cheek, especially in the region of the buccal sulcus, invariably show the presence of cocci generally arranged in pairs and often adherent to epithelial cells (*vide* Fig. 1). In perfectly healthy mouths these are often the only kind of bacteria seen on a microscopical examination. We shall refer to these cocci hereafter. Sarcinæ are often present in this region in unclean mouths and when the teeth are carious.

The space between the gums and teeth is a favorite spot for the growth of bacteria. Those which are most constantly present cannot be cultivated upon ordinary media. We believe that this is partially due to the fact that they are anaërobic, for under anaërobic

Fig. 1.

*Diplococci* in and around epithelial cell from normal healthy mouth (direct) × 1500.

(Outline of cell not very distinctly shown.)

Fig. 2.

*Bacillus maximus buccalis* from healthy mouth (direct) × 1000.

FIG. 3.

*Spirillum Sputigenum* from healthy mouth (direct) × 1000.

FIG. 4.

*Spirochæta Dentium* from healthy mouth (direct) × 1000.

conditions we have obtained a growth of several species, but we have unfortunately not yet obtained pure cultivations. The species to which we refer are leptothrix innominata, bacillus maximus buccalis, jodococcus vaginatus, spirillum sputigenum, spirochæta dentium.

The *leptothrix innominata* consists of fine interlaced threads, forming felted masses. It is probably pleomorphous, and it is possible that it represents more than one species.

The *bacillus maximus buccalis* consists of large jointed bacilli which stain of a purple color with iodine and lactic acid (*vide* Fig. 2).

The *spirillum sputigenum* consists of curved rods, which when examined in the hanging drop are motile. Some interest attaches to this bacillus, because it was formerly considered by some observers to be identical with the cholera vibrio. Its inability to grow on ordinary culture media at once distinguishes it from the latter micro-organism (*vide* Fig. 3).

The *spirochæta dentium* consists of very fine corkscrew-shaped bodies slightly pointed at the ends, and staining faintly with the aniline dyes (*vide* Fig. 4). This spirillum looks very much like the flagella seen upon other bacteria. Indeed, we are still uncertain whether some of the spirilla forms met with in the mouth are not really flagella. In some of our specimens the fine spirilla appear to be attached to the spirillum sputigenum. By examining hanging drop preparations we are, however, quite satisfied that most of the fine spirilla forms are really bacteria, for they can be observed to possess independent motility. Similar fine spirilla have also been found in the evacuation of patients suffering from cholera, and in the intestinal contents of pigs. The spirochæta dentium was found by Netter in the pus from a case of putrid empyema.

We have isolated a bacillus which agrees in its microscopical appearances with that described by Miller as the *leptothrix buccalis maxima*, and which differs from the bacillus buccalis maxima in not staining with iodine and lactic acid.

Various chromogenic bacteria have been found in the mouth. Freund isolated eighteen different species. These bacteria are interesting because they are the cause of various pigments which occur upon healthy and carious teeth and on deposits of tartar.

*Pathogenic Bacteria in the Mouth.*—We now come to the interesting question of the presence of bacteria pathogenic to the human subject in the mouths of healthy individuals.

There is abundant proof that such an occurrence is by no means uncommon. The *diplococcus of pneumonia* has been found in a large number of the cases in which it has been searched for. The best method of isolating it is to inoculate mice with saliva, and to make cultivations from the blood after death. This micro-organism is the cause of acute lobar pneumonia, and of some forms of pleurisy, cerebro-spinal meningitis, otitis, and other diseases in the human subject.

The *diphtheria bacillus* has also been found in certain cases. On several occasions one of the authors has found virulent diphtheria bacilli in the mouths of perfectly healthy individuals, or in patients convalescent from scarlet fever and with no symptoms of diphtheria. In one case it was present for several weeks in the mouth of a healthy individual.

Among the pathogenic bacteria that have been found from time to time in the mouths of healthy individuals, we may mention the staphylococcus pyogenes aureus and the streptococcus pyogenes; to the latter we shall presently revert.

The occurrence of pathogenic bacteria in the mouth is of great interest. It throws light upon the spread of disease, and shows how an apparently healthy individual may convey disease to another. This is one of the modes by which diphtheria is disseminated, and it is on this account that we meet with difficulties in attempting to eradicate the disease when it has once gained a foothold in an institution such as a large school. It, moreover, shows how carefully dental instruments should be disinfected after use. We cannot help feeling that sometimes sufficient care is not observed in this direction. It is not sufficient to rinse instruments in an antiseptic solution; they should, if possible, be placed for a few minutes in boiling water, there being no better way of sterilizing than this simple procedure.

But apart from these practical considerations, there is the theoretical question how a virulent bacterium can remain in the mouth without producing disease. This is, without doubt, due to the individual being immune. A bacterium only becomes pathogenic when it is virulent in relation to the susceptibility of the individual. The same bacterium may be harmless to one individual, and yet produce disease in another. For the production of disease two factors are necessary,—the exciting and the predisposing causes. The former, in infective diseases, is the bacterium, and the latter the susceptibility of the individual. If the individual is not susceptible, no disease is produced, and different persons vary in respect to susceptibility.

We will now discuss the question of the presence of *pathogenic*

*streptococci* in the mouth. The evidence is conflicting; some observers state that pathogenic streptococci are frequently present, others say they are seldom present, while a third series of observers maintain that a streptococcus is commonly found in the mouth which is non-pathogenic, and which is a different species to the pathogenic streptococcus.

We have made a number of observations in this direction. On making microscopical examinations of the secretion from various regions of the mouth, we have been struck with the frequent presence of masses of cocci. They are generally to be found on the tonsils and gum margin, and invariably on the mucous membrane of the buccal sulcus. We have found them present in the mouths of eighteen healthy individuals we have examined. These cocci are often found lying upon squamous epithelial cells (*vide* Fig. 1); as a rule, they are arranged in the form of diplococci, but sometimes short chains are to be seen. The individual cocci may be elongated, giving the appearance of short bacilli. Now, by making cultivations from the buccal sulcus where these cocci are always present we have invariably obtained cultivations of streptococci, which we have no doubt are the same as those seen in the microscopical preparations. That the cocci are arranged chiefly in pairs in the mouth and in chains in the cultivations is quite consistent with what we know of the morphology of streptococci.

The method we adopted is the following: A little of the scraping of the mucous membrane was removed with a platinum wire, and broth tubes were inoculated and incubated at 31° C. for twenty-four hours. At the end of this time the broth examined microscopically showed a growth consisting of diplococci, streptococci, and other bacteria. From the broth streak cultures were made on agar. Sometimes the resulting growth was a pure cultivation of streptococci, but generally other colonies also appeared; among these the most frequent were large crenated colonies of sarcinæ, especially in those cases in which a large amount of caries existed. Having obtained a cultivation on agar, pure cultivations could easily be obtained by inoculating a third series of agar tubes.

We have examined twenty-four mouths in all, sixteen with perfectly sound teeth, and eight with one or more carious teeth, and in every case streptococci were obtained in the cultivations. These observations show quite conclusively that streptococci are invariably present in the mouths of healthy individuals; they also show what care must be taken in coming to conclusions as to the signification of streptococci found in the mouth in disease. Many observers, in making bacteriological examinations of the exudation in diphtheria, lay great stress upon the presence of streptococci in addition to the diphtheria bacillus in the cultivation tubes, and they state that when many streptococci develop in the cultures the case is more severe than when the diphtheria bacillus only is present. Drs. Goodall, Card, and one of the authors have doubted this from their own observations; and the fact that streptococci are present in the normal mouth gives an explanation of these divergent results.

An exceedingly important question arises with regard to the relation of the streptococci found in the normal mouth to the streptococcus which is the cause of disease in the human subject. Is the streptococcus of the normal mouth a harmless saprophyte, which is only related to the streptococcus of disease by certain similarities, just as the hay bacillus resembles the anthrax bacillus? Or are the two micro-organisms varieties of the same species, which are capable under appropriate conditions of being mutually convertible? Can the normal streptococci of the mouth invade the body and produce disease under circumstances which lower the resistance of the body?

We will give a few examples to make these questions quite clear. In scarlet fever streptococci often invade the tissues of the tonsil, and may spread to the other tissues of the body, producing septicæmia or pyæmia. The streptococci cultivated from the resulting lesions are quite similar to the streptococci found in other septicæmic conditions, and possess a similar virulence when tested upon animals. It is a very enticing theory that the normal streptococci in the mouth have been enabled to invade the tissue of the body in virtue of the lowering of the resistance caused by the virus of scarlet fever, and that in their passage through the body they have increased in virulence. A similar example may be given in the case of puerperal fever, which is due to the invasion of the body by streptococci. Now, in the normal vagina streptococci are frequently present, and it is suggested that the lowering of resistance of the body during parturition has enabled these streptococci to invade the body,—that, in fact, a process of auto-infection has occurred. Such a view must be accepted with the greatest caution, on account of the bearing it has upon our views of the etiology and prophylaxis of infective diseases. It certainly does not agree with our knowledge of the etiology of puerperal fever, for this disease is generally conveyed by the introduction of micro-organisms from the outside by means of infected instruments or the hands of the operator. The etiology of scarlet fever is also opposed to the same view.

Besides, wounds of the mouth heal very rapidly, and this we should hardly expect if the ordinary mouth streptococcus were identical with that of disease. On the other hand, operations about the mouth may be followed by a streptococcal pyæmia. A case of this kind was under the care of one of the authors. The extraction of a tooth was followed by signs of acute septicæmia, the patient only recovering after necrosis of the alveolus of the upper jaw. Such cases may be explained on the auto-infection theory just mentioned, but another explanation is possible. Virulent streptococci may have been introduced from the outside by infected instruments, or they may have been accidentally present in the mouth.

We have already alluded to the occasional presence of virulent diphtheria bacilli in the mouth, and there is no doubt that virulent streptococci are also at times present. Several observers have undoubtedly found virulent streptococci in the mouths of healthy individuals, but this does not prove that the streptococci constantly present in the mouth are of this nature.

Our own observations have led us to incline to the view of Lingelsheim, that the normal streptococcus of the mouth is a different species to that which produces disease in the human subject.

The question is an exceedingly difficult one to decide, and an analogous question arises in the case of cholera and diphtheria. Are the vibrios met with in drinking-water different species from the cholera vibrio? and are the xerous bacillus and Hoffman's bacillus different species to the true diphtheria bacillus?

We will say a few words about the relation of Hoffman's bacillus to the diphtheria bacillus, for we believe the question to be somewhat analogous to that concerning the streptococcus. Hoffman's bacillus is not infrequently found in normal mouths, and has, no doubt, in many instances been mistaken for the true diphtheria bacillus. It resembles it in cultivations, and to some extent microscopically, especially in old cultivations, when it may become clubbed like the diphtheria bacillus. It is not, however, pathogenic to animals, and can be distinguished from the diphtheria bacillus by the careful comparison of cultures made under similar conditions. It has never been converted into the true diphtheria bacillus by artificial means, and is probably a different species, belonging, however, to the same group. We have already stated that the true pathogenic diphtheria bacillus may be present in the mouths of healthy individuals.

Now to understand the difficulties in distinguishing the normal streptococcus of the mouth from that of disease, we must say something of the varieties of streptococci found in various diseases in the human subject. Streptococci are the cause of erysipelas, pyæmia, puerperal fever, and a variety of other septic affections. The streptococci obtained from different cases of these diseases agree with one another in their main features, and have been grouped together by Lingelsheim into one species, called by him the streptococcus longus.

The general characters of this streptococcus are the following: It grows best at 37° C., but will grow at the ordinary temperature of the air. On agar and gelatin the colonies are minute and semi-transparent, the latter medium is not liquefied. The growth in broth is rather characteristic; flocculent masses stick to the sides and fall to the bottom of the tube, while the rest of the broth remains clear and transparent. A slight amount of acidity is produced in the cultivations. The microscopical appearance of the broth cultivations is characterized by the length of the chains, some consisting of as many as forty members. Hence the name streptococcus longus. In other media the chains are often much shorter, and in the tissues of infected animals only diplococci forms may be met with.

These are the main characters of the streptococcus longus, but cultural and microscopical differences are to be observed in the micro-organisms obtained from different sources. For example, broth may be rendered uniformly turbid, instead of presenting the characteristic growth above described. Differences in virulence in animals have, however, been looked upon as the most important point of distinction between pathogenic streptococci obtained from different sources. The streptococcus of erysipelas has been distinguished from the streptococcus pyogenes by being more virulent to rabbits than to mice.

The test of virulence is, however, very fallacious, as will be shown by the following experiments. We obtained streptococci from the following sources: (1) a case of suppurating knee-joint in a man; (2) a fatal case of pyæmia in a child; (3) a severe case of phlegmonous erysipelas in a woman; (4) an abscess from a horse. One cubic centimetre of a twenty-four hours' old broth from each case was injected into the peritoneal cavity of a rabbit, and a large loopful of a twenty-four hours' old agar cultivation from each case was inserted under the skin of a mouse. In none of the cases obtained from the human subject were the animals affected, while the rabbit, inoculated with the cultivation from the horse, died of septicæmia in twenty-four hours, and the mouse in two days.

Almost all observers have noted a great difference in the virulence of streptococci to animals even when taken from apparently similar cases in the human subject.

Marmorek has shown that streptococci obtained from various diseases in the human subjects, although differing initially in pathogenic effects on animals, can, nevertheless, be raised to the same pitch of virulence by repeated passages and cultivation in special media. He consequently looks upon the streptococci pathogenic to the human subject as all simple varieties of the same species which can be converted into one uniform type by appropriate means.

Lingelsheim was the first to point out that the streptococcus obtained from the normal mouth differed from the streptococcus longus in three points. It was not pathogenic to rabbits or mice; it rendered broth turbid, and the chains in this latter medicine were shorter than those of the streptococcus longus; and it caused slight liquefaction of gelatin. He considered it a distinct species and called it the streptococcus brevis.

His observations have been confirmed by some investigators, while others have disputed his view. We have made a careful series of cultivation and inoculation experiments with streptococci obtained from the mouths of three healthy individuals, and have compared them with streptococci obtained from cases of pyæmia and phlegmonous erysipelas.

The details of the experiments we will not weary you with, but will only give you the main results. The streptococcus from the normal mouth differs from the streptococcus longus in the following points: (1) It is non-pathogenic when tested upon rabbits and mice; but we have already stated that this test of virulence in the case of streptococci is not very conclusive. (2) It produces a uniform turbidity in broth cultivations. (3) It clots milk and produces much more acid than the streptococcus longus. (4) The individual cocci are smaller, and the chains, especially in broth cultivations, shorter. The length of the chains is not always a reliable criterion. Sometimes in the impure cultivation obtained from the mouth the chains are very long, and this is probably due to the medicine being altered in composition by the other bacteria present.

We have not been able to confirm the observations of Lingelsheim that gelatin is liquefied.

Slight differences have been observed in the streptococci we have examined. Two of them grow more slowly in the cold than the other and present somewhat different microscopical appearances. The cultivations we are passing round illustrate these different points (*vide* also figures).

The conclusions which we draw are these: The streptococcus occurring normally in the mouth agrees with the streptococcus brevis of Lingelsheim, and can be distinguished from the streptococcus of disease by its biological and morphological characters. It must be looked upon as a distinct species for the present, although ultimately this view may be proved incorrect, for it is possible that further researches may enable us to convert the streptococcus brevis into the streptococcus longus. This, however, has hitherto not been accomplished. We think that the discrepancies of different observers who have investigated the streptococci of the mouth are partially due to the fact that the streptococcus longus is sometimes accidentally present and has been mistaken for the normal streptococcus of the mouth.

## Figures and Tables

**Fig. 1. f1:**
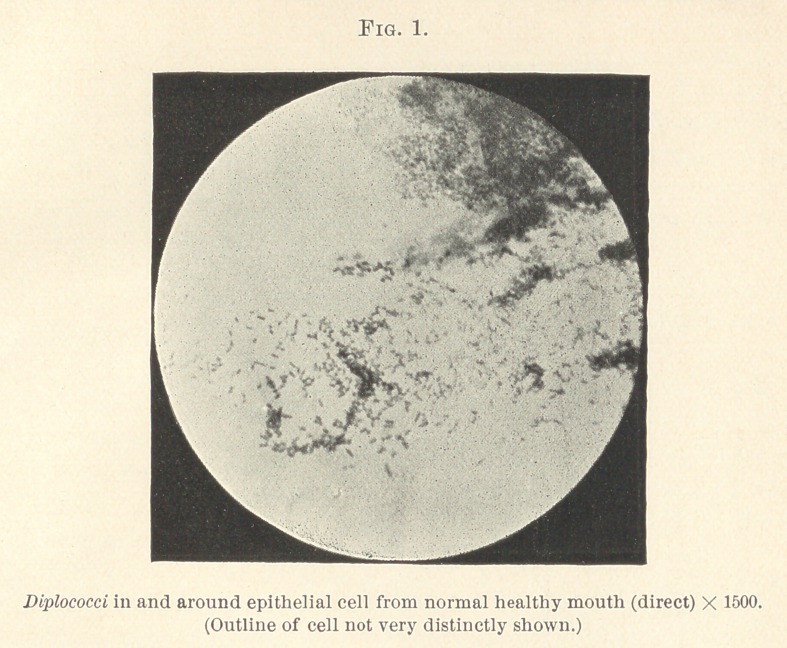


**Fig. 2. f2:**
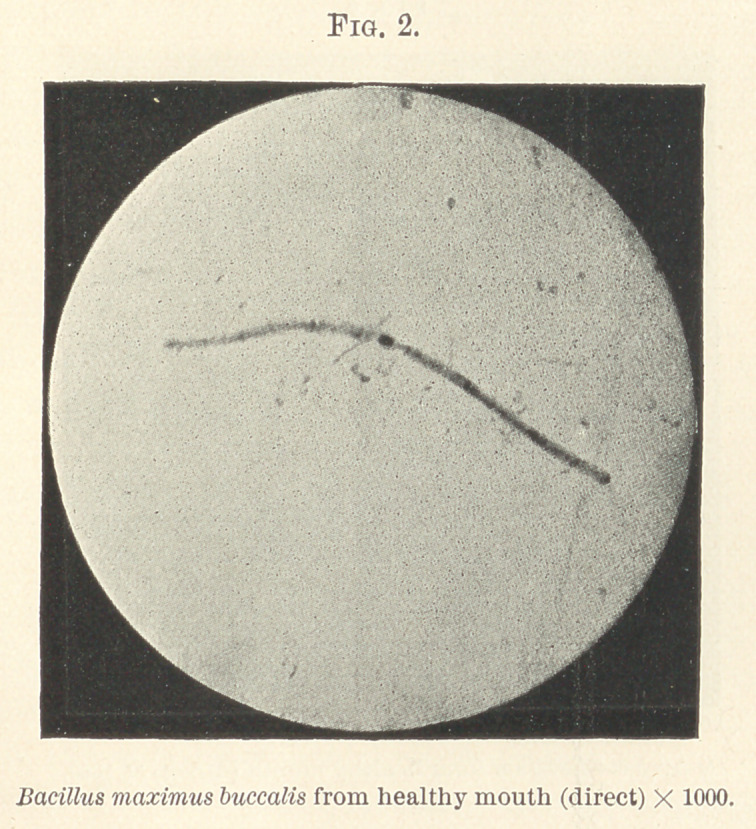


**Fig. 3. f3:**
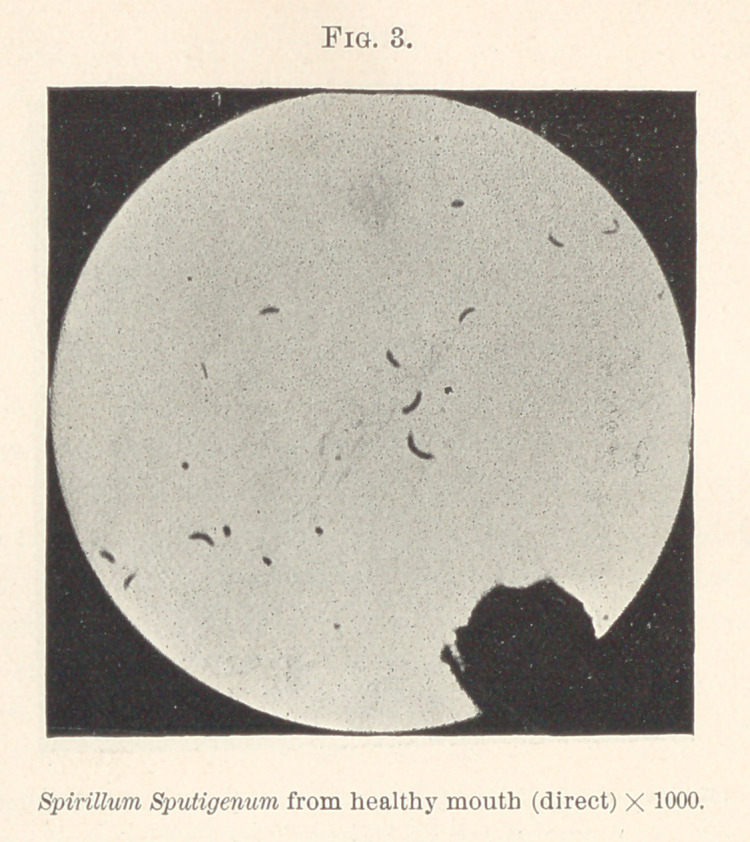


**Fig. 4. f4:**